# Self-Healable, Antimicrobial and Conductive Hydrogels Based on Dynamic Covalent Bonding with Silver Nanoparticles for Flexible Sensor

**DOI:** 10.3390/polym17010054

**Published:** 2024-12-29

**Authors:** Te Qi, Xuefeng Liu, Nan Zheng, Jie Huang, Wenlong Xiang, Yujin Nie, Zanru Guo, Baixue Cai

**Affiliations:** 1 College of Chemistry, Chemical Engineering and Environmental Science, Minnan Normal University, Zhangzhou 363000, Chinahj1745@mnnu.edu.cn (J.H.); wenlongx2290@mnnu.edu.cn (W.X.);; 2 Chongqing Academy of Metrology and Quality Inspection, Chongqing 401120, China

**Keywords:** hydrogel sensor, self-healing, silver nanoparticles, antimicrobial properties, acylhydrazone bonds

## Abstract

Dynamic hydrogels have attracted considerable attention in the application of flexible electronics, as they possess injectable and self-healing abilities. However, it is still a challenge to combine high conductivity and antibacterial properties into dynamic hydrogels. In this work, we fabricated a type of dynamic hydrogel based on acylhydrazone bonds between thermo-responsive copolymer and silver nanoparticles (AgNPs) functionalized with hydrazide groups. The hybrid hydrogels exhibited sol–gel transition, self-healable, injectable and thermo-responsive abilities. The self-healing efficiency was over 92%. Moreover, the hydrogel displayed antimicrobial properties and high conductivity (6.85 S/m). Notably, the fabricated hydrogel-based sensors exhibited strain and temperature sensing (22.05%/°C) and could detect human motion and speech, and electrocardiographic (ECG) and electromyography (EMG) signals. Overall, this work provides a simple strategy to synthesize AgNPs-based dynamic hydrogels with multi-functions, and the hydrogels may find potential applications in antibacterial wearable electronics, health monitoring and speech recognition.

## 1. Introduction

As one of the soft materials with 3D network structure storing large amounts of water, hydrogels have tremendous applications ranging from drug delivery [1], wound dressings [2], soft robotics [3,4], flexible electronics [5,6] and so on. Among them, there is an increasing interest in hydrogel-based flexible electronics [6], because hydrogels possess softness comparable to biological tissues and can provide an environment similar to that of extracellular matrix [7,8]. Thus, hydrogel-based flexible sensors could contact with target tissue seamlessly in comparison with traditional rigid devices, which enable the sensors to record motion and electrophysiological (EP) or other physiological signals [9]. As for hydrogel sensors, conductive hydrogels were usually employed, as they converted the mechanical or physiological signals into electrical signals [10]. Conductive hydrogel contains a hydrogel matrix and conductive component [6]. However, it is hard for hydrogel sensors to avoid the mechanical damage by external pressure when they are used [11]. To extend the service life of hydrogel sensors and reduce waste, it is of great significance to develop self-healable hydrogel matrix capable of autonomously repairing damage without any external intervention.

Self-healable hydrogels would form by the introduction of dynamic covalent bonds (DCBs) or noncovalent bonds [12]. Compared with noncovalent bonds, dynamic covalent bonds can dissociate, recombine and exchange reversibly in hydrogel networks [13], which integrate the reversibility of noncovalent bonds and the stability of covalent bonds [12,13]. DCBs serving as crosslinking in hydrogel were concerned. To date, several DCBs such as phenylboronate esters [14], disulfide bonds [15] and acylhydrazone [16] have been used to prepare self-healable hydrogels [17,18]. Among them, acylhydrazone bonds, formed from the condensation of acylhydrazine and aldehyde/ketone, were concerned due to their easy designability and thermodynamic stability [19]. Recently, we and other researchers [12,13,19,20,21,22] have prepared hydrogels by using acylhydrazone bonds as crosslinking. The hydrogels exhibited self-healing ability without any external stimuli and possessed injectable and sol–gel transition properties. However, those hydrogels were mainly used as drug delivery vehicles and cell culture scaffolds in view of their biocompatibility [12,19]. Acylhydrazone-based hydrogels were rarely explored for the application of flexible sensors.

For hydrogel sensors, it is necessary to endow hydrogels with improved conductivity. Embedding conductive fillers, such as ionic liquids [23], semiconductors [24], carbon materials [25] and nano-metals [26,27,28], is considered a facile and feasible method to enhance the conductivity of hydrogels [29]. Among these conductive fillers, silver nanoparticles (AgNPs) could be regarded as a potential choice for the conductor due to its low toxicity, broad-spectrum antibacterial properties and high conductivity [30]. A hydrogel sensor with antibacterial ability will reduce bacterial infection when it is applied to the human body. Han et al. [31] introduced the lignin–silver hybrid nanoparticles into the polyvinyl alcohol matrix and found that the hydrogel has sensitivity for compression. Fan et al. [32] dispersed AgNPs-attached CNCs in the hydrogel matrix and obtained the hydrogels with monitoring for various human movements. Wang et al. [33] prepared hydrogels with AgNPs for antibacterial strain sensors. However, AgNPs in those hydrogels were mainly dispersed in the hydrogel network rather than participating in the formation of the network [30,31,32,33]. AgNPs might have dropped from the carrier and agglomerated in the network after repeated stretching and compression. In addition, the hydrogels with AgNPs were mainly used to detect the motion [34,35], and the monitoring for other physiological signals such as temperature were rarely examined. When AgNPs have functional groups and form acylhydrazone bonds with temperature-responsive polymers, it is anticipated that the AgNPs will immobilize in the network hydrogels, and the hydrogel not only possesses self-healable, antibacterial, injectable and sol–gel transition properties but also exhibits temperature sensing due to the thermo-responsive hydrogel network.

**Scheme 1:**
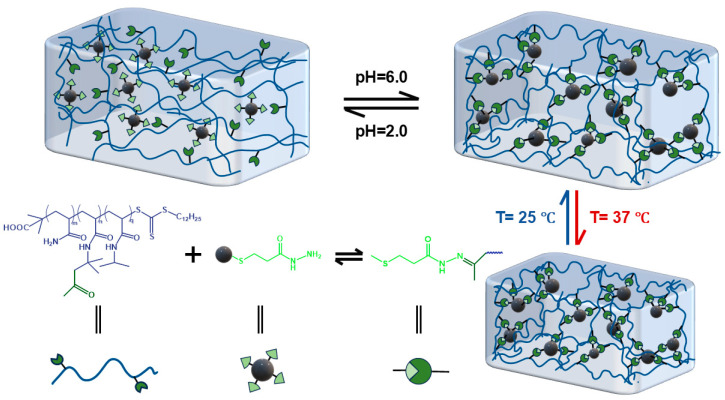
Schematic of the fabrication of self-healable and sol–gel transition hydrogels based on AgNPs.

In this paper, we report a type of self-healable, injectable and antimicrobial conductive hydrogel possessing motion and temperature sensing based on a thermo-responsive copolymer and AgNPs functionalized with hydrazide groups, as illustrated in Scheme 1. The thermo-responsive copolymer poly[(*N*-isopropylacrylamide)-co-(diacetone acrylamide) -co-poly[(acrylamide)] (P(NIPAM-co-DAAM-co-AM)) was synthesized by reversible addition–fragmentation chain-transfer (RAFT) polymerization. AgNPs with hydrazide groups were obtained by surface functionalization and hydrazinolysis. The conductive hydrogels were generated by mixing the copolymer and AgNPs via the formation of acylhydrazone bonds between ketone groups and hydrazides (Scheme 1). Based on dynamical acylhydrazone crosslinking, the obtained hydrogels exhibited sol–gel transition, self-healable, injectable and thermo-responsive abilities. Moreover, the hydrogel displayed antimicrobial properties, and the fabricated hydrogel-based sensors exhibited strain and temperature sensing. The hydrogel can also be used as an electrode for ECG and EMG signals detection. With these attractive characteristics, the dynamic hydrogels may find potential applications in antibacterial wearable electronics, health monitoring and speech recognition.

## 2. Materials and Methods

### 2.1. Materials

Silver nitrate (AgNO_3_, 99.8%, Aladdin Co., Ltd., Shanghai, China), methyl 3-mercaptopropionate (98%, Aladdin) and acrylamide (AM, >98%, Aladdin Co., Ltd.), sodium borohydride (NaBH_4_, 98%, Aladdin Co., Ltd.), hydrazine hydrate monohydrate (>98%, Aladdin Co., Ltd.), diacetone acrylamide (DAAM, 98%, TCI) and azodiisobutyronitrile (AIBN, 99%, Aladdin Co., Ltd.) were used as received. *N*-isopropyl acrylamide (NIPAM, 98%, TCI) was purified by recrystallization. A total of 4 M NaOH and HCl was used to adjust the pH of solutions. 2-(1-carboxy-1-methyl-ethylsulfanylthio- -carbonylsulfanyl)-2-methylpropionic acid was used as a chain transfer agent (CTA) and was synthesized following a previous report [36]. All other chemicals were analytical grade and used as received.

### 2.2. Preparation of Temperature-Sensitive Polymer Containing Ketone Groups

The copolymer was prepared according to our previous report [37]. NIPAM (4.0 g, 35.40 mmol), AM (0.718 g, 10.10 mmol) and DAAM (0.855 g, 5.06 mmol) were added to a two-necked vial with 30 mL dimethyl sulfoxide (DMSO), and then nitrogen was bubbled into the solution for 30 min to remove oxygen. The chain transfer agent CTA (922.0 mg, 0.25 mmol) and AIBN (2.1 mg, 0.013 mmol) were added with the protection of N_2_. The solution was stirred continuously for another 5 min and placed in an oil bath at 70 °C for polymerization. After 24 h, a large amount of ether was added dropwise to obtain white precipitate. The product was collected and redissolved in DMSO and was reprecipitated into excess ether. This purification cycle was repeated twice. The resulting products were freeze-dried for 24 h to yield the objective copolymer, denoted as P_70_, where “P” means the polymer and the subscript “_70_” is the mole ratio of NIPAM to the total moles of the copolymer. The molar ratio of NIPAM, AM and DAAM in copolymer was 70:20:10.

### 2.3. Preparation of AgNPs Containing Hydrazide Groups

Three types of AgNPs containing hydrazide groups were prepared (Table S1). A typical experiment of AgNPs1 was as follows: methyl 3-mercaptopropionate (0.1200 g) dissolved in methanol (10 mL) was added dropwise into methanol (100 mL) with 0.85 g of AgNO_3_ under high-speed stirring with a shading condition. After stirring for 4 h, NaBH_4_ (0.5630 g) was slowly added to the solution (the solution immediately turned black). After the addition of NaBH_4_, the mixture was stirred for another 4 h. Then, hydrazide hydrate (0.2300 g) was added and the solution refluxed at 70 °C for 12 h. Subsequently, AgNPs were obtained by centrifugation at 10,000 rpm for 5 min. The remaining solid was washed with anhydrous ethanol three times to remove any impurities. AgNPs containing hydrazides were dried in a vacuum oven at 50 °C for 24 h. Pure AgNPs were synthesized by adding NaBH_4_ (0.5630 g) into AgNO_3_ (0.85 g) and methanol (100 mL), and used as control.

### 2.4. Fabrication of Conductive Hydrogels

The conductive hydrogels were prepared by a simple mixing of the copolymer P_70_ solution and AgNPs solutions, where the number of hydrazide groups from AgNPs was equivalent to that of the ketone groups of the copolymer. The hydrazide groups per gram of AgNPs1, AgNPs2 and AgNPs3 were 1.14 mmol, 1.70 mmol and 2.24 mmol, respectively, which were determined by TGA (discussed in Section 3.2). The ketone groups per gram of P_70_ were 0.947 mmol, based on the unit’s molar ratio (15.3:10.7:74.0) calculated by ^1^H NMR spectra (discussed in Section 3.1). The solid content was kept at 10 wt%. The obtained hydrogels based on AgNPs1, AgNPs2 and AgNPs3 were labeled as HAg1, HAg2 and HAg3, respectively. The compositions of hydrogels are listed in Table S3. The procedure for the fabrication of HAg1 was as follows: 1.66 g AgNPs1 was dispersed into 32.94 g D. I. water by sonication for 30 min, then 2.00 g copolymer was dissolved in the dispersion by stirring. The pH of the mixture solution was tuned to ca. 6.0 by using 4 M HCl and 4 M NaOH. The viscous solutions became hydrogels after ca. 1 h at room temperature. The hydrogels were kept at room temperature for 24 h before testing. The preparation procedures for HAg2 and HAg3 were similar to that of AgNPs1.

### 2.5. Self-Healing and Injectable Properties of the Hydrogel

For the self-healing testing, one disk-shaped hydrogel was cut into two halves. Thereafter, two semicircular hydrogels rejoined for 15 min at room temperature. To calculate the self-healing efficiency, the tensile stress–strain curves of the rectangle-shaped hydrogels (40 mm × 10 mm × 10 mm) and the self-healed hydrogels were measured. The self-healing efficiency is defined as the ratio of the stress at fracture to the original stress. The mean values were calculated from at least three replicate experimental data.

The injectability experiment of the hydrogel was implemented by extruding HAg1 from a syringe.

### 2.6. Antibacterial Test of Hydrogels

*Staphylococcus aureus* (*S. aureus*, ATCC 25923) was used as the tested strain, and the trypticase soy agar (TSA) nutrient medium was used to cultivate it at 37 °C for 24 h. The cylindrical hydrogels (the diameter was 1.4 cm) were transferred to a Luria–Bertani (LB) agar plate covered with fresh *S. aureus* suspension. After incubation at 37 °C for 24 h, the diameter of the antibacterial circle was measured with vernier calipers, and the average value was obtained from at least three replicate experimental data.

The morphology changes of microorganisms on hydrogels were examined with SEM. A total of 1 mL of fresh *S. aureus* suspension (1 × 10^6^ CFUs/mL) was incubated with the hydrogels at 37 °C for 6 h. The hydrogel samples were washed with PBS 3 times and then fixed with 2.5% glutaraldehyde solution for 4 h. The samples were dehydrated in a graded ethanol series from 50% to 100% each for 10 min, and the samples were dried in air at room temperature. They were observed with SEM for microbe morphology changes after coating with gold.

### 2.7. Characterization

^1^H NMR analyses were conducted on a Brucker 300 MHz spectrometer (Brucker, Wissembourg, France) at room temperature, and D_2_O was used as the solvent.

Fourier transform infrared (FT-IR) spectra were obtained on a Nicolet MX-1E FTIR spectrophotometer(Thermo Electron Corporation, Waltham, MA, USA). The dried samples were mixed with KBr and compressed into films.

Gel permeation chromatography (GPC) was performed using HCC-8320GPC (Higashima Co., Ltd., Tokyo, Japan) with equipped with a TSK GEL Super AWM-H column and refractive index detector. DMF was used as the eluent (the flow rate is 0.4 mL min^−1^). Mono-dispersed polymethyl methacrylate was used as the standard.

SEM observation was conducted on a JSM-6510 (Japan Electronics Co., Ltd, Tokyo, Japan). The hydrogel samples were frozen by liquid nitrogen and then dried by vacuum freeze-drying.

X-ray diffraction (XRD) patterns were recorded using a RigakuDMAX 2200 (Rigaku corporation, Tokyo, Japan) with Ni-filtered CuKα radiation in the scanning range of 30° to 90°, at 40 kV and 40 mA X-ray power.

The conductivity of hydrogels was measured at room temperature by a four-probe instrument SB1201 (Shanghai Qianfeng Electronic Instrument Co., Ltd., Shanghai, China). The round hydrogels (5 mm thickness, 30 mm diameter) were used for the conductivity test. The mean values and errors were calculated from at least three independent samples for each type of hydrogel.

Thermogravimetry analysis (TGA) was conducted on a Diamond TG/DTA thermal analysis system (PerkinElmer, Hopkinton, MA, USA). Samples were heated in a flow of N_2_ (20 mL min^−1^) from room temperature to 800 °C at a heating rate of 10 °C min^−1^.

Rheological characterization was performed on a Physica MCR 302 (Anton Paar, Graz, Austria) rheometer by the flat plate mode with a diameter of 25 mm. The experiments were conducted by the stress- or strain-controlled mode. For the modulus changing upon temperature, the temperature increasing rate is 0.2 °C min^−1^.

The tensile strain–stress tests of the hydrogels (40 mm × 10 mm × 10 mm) were conducted on a universal testing machine BJ-SPLZ (Guangzhou Biaoji Packaging Equipment Co., Ltd., Guangzhou, China) fitted with a 20 N load cell at room temperature. The tensile speed was 50 mm/min.

Sensor tests were recorded on a digital multimeter TH2829A LCR meter (Changzhou Tonghui Electronics Co., Ltd., Changzhou, China). The gauge factor (GF) used to evaluate the sensitivity of sensors was calculated by the following equation: GF = ΔR/R_0_/ε, where ΔR and R_0_ are the resistance difference after stretching and the initial resistance, and ε is the tensile strain of hydrogels. For the fabrication of the hydrogel-based sensor, both ends of the hydrogel (20 × 10 × 2 mm^3^) were winded with copper wires serving as the current collectors. The other ends of copper wires were connected to the LCR meter for recording the resistance. The test was carried out by stretching with a universal testing machine or attaching to the subjects’ bodies, such as the wrist and throat.

Electrocardiographic (ECG) and electromyography (EMG) signals were recorded by a commercial ECG and EMG monitor with three electrodes. Round hydrogels (1.5 mm thickness, 20 mm diameter) were used as electrodes and were directly adhered to the skin.

## 3. Results and Discussion

### 3.1. Fabrication and Structural Characterization of Temperature-Responsive Polymer

To obtain the polymer with a desirable molecular weight, RAFT polymerization was employed to prepare the copolymer [38]. According to our previous report [37], the cloudy point of the temperature-responsive polymer containing ketones could be well tuned by variation of the components of NIPAM, DAAM and AM units. NIPAM, DAAM and AM three monomers with a molar ratio of 70: 10: 20 were chosen to form the copolymer P_70_, as its cloudy point was close to the human body temperature (37 °C) [19,37]. The structure of P_70_ was confirmed by ^1^H NMR spectra (Figure S1a). The signals at 0.9~1.2 ppm and 3.7~4.0 ppm were assigned to the protons from NIPAM units [39]. The chemical shift peaks of −(C*H*_3_)_2_C− on DAAM units and the protons on the main chain of P_70_ occurred at 1.2~1.8 ppm. The peaks of 1.8~2.3 ppm were ascribed to the protons from −C*H*− of the main chain and −C*H*_3_ on DAAM units. The peak at 2.8–3.3 ppm was assigned to −C*H*_2_− protons on DAAM units [40]. The signals of hydrogen protons were in agreement with our previous reports [37,39]. According to our previously reported equation [37,39], the unit ratios of P_70_ were calculated based on the integration of the proton peaks in the corresponding units. The molar ratio of AM, DAAM and NIPAM units was 15.3:10.7:74.0, which was slightly different than the feeding ratio due to the steric effect [37]. GPC was performed to measure the molecular weight (M_n,GPC_) and polydispersity index (PDI) of P_70_ (Figure S1b and Table S2). It was found that the M_n,GPC_ was close to the theoretical value, and its PDI is 1.483, indicating a copolymer with a controllable molecular weight and molecular weight distribution [41].

PNIPAM is a typical temperature-responsive polymer that can be converted from soluble to insoluble as the temperature rises [39,42]. To confirm the temperature responsiveness of P_70_, the apparent phenomenon of P_70_ solution was observed during the changing of the temperature. As shown in Figure S2, the solution is colorless and transparent at 25 °C, while the solution appeared milky white when the temperature increased to 40 °C, which indicated that the prepared copolymer had a temperature-responsive property [33]. To further determine the cloudy point of P_70_, the transmittance of the solution at 600 nm along with the temperature was tested. As shown in Figure S2, the transmittance of the solution started to decrease when the temperature was increased to 35 °C, and the transmittance dropped to nearly 0% as the temperature increased to 37.5 °C. The cloudy point of P_70_ was 36.3 °C, which is the midpoint temperature at the curve of transmittance with increasing of the temperature. The cloudy point of P_70_ was close to that of the human body temperature [43].

### 3.2. Synthesis and Characterization of AgNPs Hybrids Containing Hydrazide Groups

**Figure 1:**
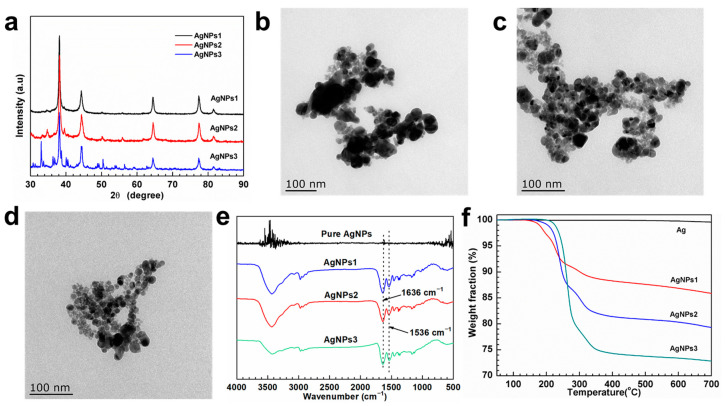
(**a**) XRD pattern of AgNPs with hydrazide groups; TEM images of AgNPs1 (**b**), AgNPs2 (**c**) and AgNPs3 (**d**); (**e**) FT-IR spectra of pure AgNPs and AgNPs hybrids; (**f**) TGA traces for pure AgNPs and AgNPs hybrids.

AgNPs hybrids with hydrazide groups were synthesized by surface functionalization and hydrazinolysis. XRD was performed to confirm the formation of AgNPs hybrids. As shown in Figure 1a, five diffraction peaks appeared in the XRD pattern at 2θ positions 38.1°, 44.2°, 64.4°, 77.4° and 81.5°, which are assigned to [1 1 1], [2 0 0], [2 2 0], [3 1 1] and [2 1 1] planes of the face-centered cubic (fcc) of AgNPs [44,45] in comparison with the JPDS card (no.: 04-0783). It also was found that there were other miscellaneous diffraction peaks with the increase in hydrazide bonds (such as AgNPs3), which might be attributed to too much hydrazide component having a negative effect on the crystallization of AgNPs3.

TEM was employed to visualize the morphology of AgNPs directly. As shown in Figure 1b–d, AgNPs containing hydrazide bonds exhibited a spherical shape and became smaller and more uniform in size distribution at the increasing of methyl 3-mercaptopropionate. This may be explained by AgNPs being wrapped with methyl 3-mercaptopropionate through a chemical bond between S ions and Ag atoms, and the increasing of the 3-mercaptopropionate ratio made more compounds on the AgNPs surface, which led to AgNPs hybrids being dispersed more homogeneously in the solution [46]. Therefore, the AgNPs size could be adjusted by changing the molar ratio of the compound to AgNO_3_.

To check that hydrazide groups were introduced on AgNPs, FT-IR spectroscopy was employed to characterize the AgNPs hybrids. As shown in Figure 1e, AgNPs hybrids showed strong peaks at 1636 cm^−1^ and 1536 cm^−1^ in comparison with that of pure AgNPs, which were ascribed to the characteristic absorption band of C=O from ketone carbonyl groups and N-H of hydrazides [19,37,47], respectively. This suggests that hydrazide groups attached on the AgNPs surface. To determine the relative number of hydrazide groups on AgNPs, TGA measurements were performed. As given in Figure 1f, the weight loss of AgNPs under a nitrogen atmosphere happened in the temperature range from ca. 150 °C to ca. 380 °C, resulting from the decomposition of the organic component on AgNPs. One can find that the weight percentages of the organic component in AgNPs1, AgNPs2 and AgNPs3 are 13.7 wt%, 20.4 wt% and 26.9 wt%, respectively, providing additional evidence of hydrazide groups on AgNPs. The mole number of hydrazide groups per gram of AgNPs could be calculated by dividing the molecular weight of the organic component. The values for AgNPs1, AgNPs2 and AgNPs3 are 1.14 mmol, 1.70 mmol and 2.24 mmol, respectively, which suggests that the content of hydrazide groups on AgNPs can be regulated by feeding ratios.

### 3.3. Preparation and Characterization of Dynamic Hydrogels with AgNPs

**Figure 2:**
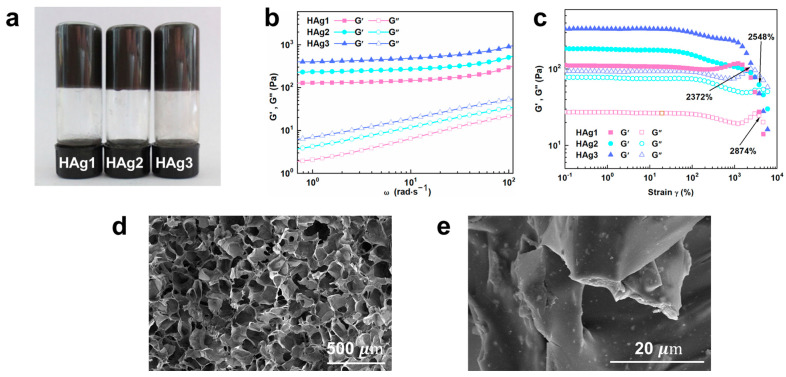
(**a**) Image of the obtained hydrogels; (**b**) *G′* and *G″* of the hydrogels as functions of frequency (ω); (**c**) Strain amplitude sweep of the hydrogels with a fixed frequency of 1 Hz at 25 °C; (**d**) SEM image of HAg1 and (**e**) its magnified SEM image on skeleton.

The dynamic acylhydrazone can be formed between ketone and hydrazide [19,39], which served as the crosslinking for hydrogels. Thus, the copolymer P_70_ containing ketone groups was mixed with AgNPs hybrids, resulting in the formation of dynamic hydrogel HAg1, HAg2 and HAg3. As shown in Figure 2a, the mixture of P_70_ and AgNPs hybrids displayed as gels and could support their weight in a vial when the pH of the solutions was adjusted to ca. 6. To confirm the formation of the hydrogels, dynamic rheology measurements were performed. Figure 2b shows the dynamic modulus versus the frequency at a 1% strain at 25 °C. One can find that the storage modulus (*G′*) of the three hydrogels was consistently higher than their loss modulus (*G″*) in the tested frequency range, indicative of the formation of hydrogels [13,19]. In addition, *G′* of HAg1, HAg2 and HAg3 were ca. 126 Pa, ca. 227 Pa and ca. 395 Pa, i.e., *G′* increased gradually with the increment of hydrazide groups on the AgNPs surface. This should be attributed to the greater the number of hydrazide groups in one nanoparticle is, the greater the amount of polymer chains bonded together, forming a more robust network and leading to a higher modulus.

In addition, strain amplitude sweeps for obtained hydrogels were measured at a fixed frequency of 1 Hz at 25 °C. As illustrated in Figure 2c, the *G′* of the hydrogels was greater than *G″* at the initial strain region. When the strain reached a critical value, *G″* overlapped with *G′*, suggesting that the network of the hydrogel was destroyed. The critical strain values of HAg1, HAg2 and HAg3 were 2877 ± 13%, 2545 ± 9% and 2373 ± 14%, respectively, which indicates that the hydrogels could withstand large deformation. However, the critical strain value decreased on the contrary, though the *G′* increased from HAg1 to HAg3. This should be attributed to the chains in the network gradually becoming tight due to the increment of hydrazide groups in one nanoparticle, which would increase the stiffness of the hydrogel and concomitantly might reduce the movement of polymer chains. This led to the disruption of hydrogels at a lower strain.

SEM was carried out to observe the microstructure of the hydrogels. As shown in Figure 2d, HAg1 displayed a uniform three-dimensional porous structure, further indicating the formation of hydrogel between AgNPs hybrids and copolymer. Moreover, AgNPs hybrids were uniformly distributed in the skeleton of hydrogel (Figure 2e). HAg2 and HAg3 also showed similar porous structures (Figure S3). However, the pore size increased, and its skeleton became thicker from HAg1 to HAg3 (Figure 2d and Figure S3), which further indicated that the increment of hydrazide groups in nanoparticles would react with more polymer chains. In order to further verify the formation of hydrogels with AgNPs, the prepared hydrogels were characterized by elemental analysis. The EDS spectra of the hydrogels is shown in Figure S4. One can find that the hydrogels contained C, N, O, S and Ag elements, which originated from AgNPs hybrids and the copolymer. However, it should be noted that the content of Ag gradually decreased from HAg1 to HAg3. This result should be attributed to the feeding ratio of AgNPs hybrids in hydrogel decreasing (Table S3) with the increasing of hydrazide groups on the AgNPs surface due to the ketone groups remaining constant.

To confirm the formation of hydrogels via acylhydrazone crosslinking, FT-IR was performed to analyze the precursors and hydrogel. The characteristic absorption peak of the C=O of ketones in the polymer appeared at 1720 cm^−1^ (Figure S5). However, when the AgNPs1 were mixed with the copolymer to form HAg1, the characteristic absorption peak of C=O at 1720 cm^−1^ disappeared, while the characteristic absorption of the C=N bond of acylhydrazone appeared at 1665 cm^−1^ [37], indicating the formation of the acylhydrazone crosslinking in the network. To demonstrate the formation of hydrogels based on AgNPs, the hydrogels were analyzed by XRD. As shown in Figure S6, five diffraction peaks of AgNPs appeared at 2θ of 38.1°, 44.2°, 64.4°, 77.4° and 81.5°, consistent with that of AgNPs hybrids. This result indicated that AgNPs in hydrogels showed a face-centered cubic structure, and AgNPs were not destroyed during the formation of hydrogels. In addition, AgNPs in hydrogels remained stable even after storing at room temperature for 1 month, as it was found that XRD patterns of the hydrogels were similar to that of initial hydrogel samples (Figure S7).

### 3.4. Self-Healing and Injectable Properties of the Hydrogels

**Figure 3:**
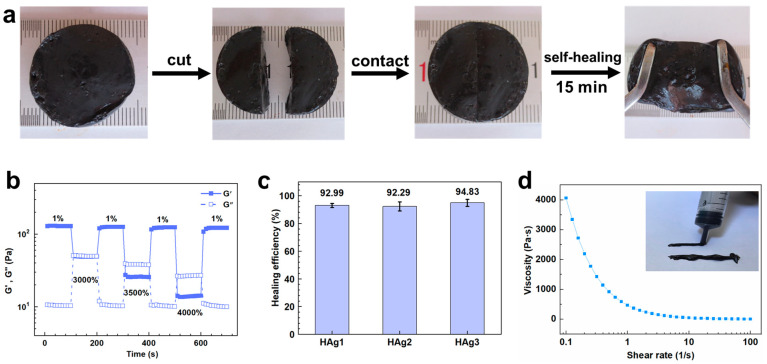
(**a**) Self-healing process of HAg1; (**b**) Step-strain measurements of HAg1 at 25 °C; (**c**) The self-healing efficiency of the hydrogels; (**d**) Viscosity and shear-thinning behavior of HAg1. The inset image was HAg1 injected with a conventional syringe.

According to previous reports [19,37], hydrogels constructed with acylhydrazone bonds have self-healing properties. Therefore, the self-healing properties of hydrogels were visually examined. As shown in Figure 3a, a circular hydrogel HAg1 was cut into half. Then, the two semicircle gels were spliced together for 15 min without any external intervention. It was found that they could heal completely and be gently stretched with forceps, indicating the self-healing of the hydrogel. Moreover, step-strain measurements were carried out to verify the self-healing property. As shown in Figure 3b, when the applied oscillatory shear strain was stepped from 1% to 3000% and maintained for 100 s, the *G″* slightly surpassed *G′*, while they immediately restored to their initial values after the strain returned to 1%. Similarly, when a larger strain (3500% and 4000%) was applied to the hydrogel, *G″* became greater than *G′*, indicating that the network was destroyed and appeared as a liquid-like state. However, *G′* and *G″* were quickly recovered when the strain returned to 1%, which implies the automatic repair of the hydrogel network [11,30]. The self-healable ability of hydrogels should be attributed to the reversibility of acylhydrazone bonds (Scheme 1), which could reform at the interfaces of broken hydrogels [15,16,19]. Furthermore, tensile stress–strain tests were conducted to assess their self-healing efficiency. The tensile stress–strain curves before and after self-healing hydrogels are shown in Figure S8. The self-healing efficiencies of the three hydrogels were 92.99%, 92.29% and 94.83% (Figure 3c), respectively, which proved that the hydrogels have high self-healing efficiency and their mechanical properties could be recovered after being damaged. In addition, the hydrogels also exhibited high self-healable abilities after storing at room temperature for two weeks. As shown in Figure S9, the self-healing efficiencies were still over 85%. However, it should be pointed out that the fracture stresses of the hydrogels were slightly higher than those of freshly prepared samples (Figure S9a), while the self-healing efficiencies decreased slightly (Figure S9b). This should be attributed to the evaporation of water, though they were kept under seal, reducing the movement of polymer chains.

As mentioned above, the hydrogel network could be disrupted at a large shear strain, implying the shear-thinning behavior of hydrogel. Thus, the variation of the gel’s viscosity with the increasing of the shear rate was measured. As shown in Figure 3d, the viscosity of HAg1 decreased with the increasing of the shear rate, which resulted from the disruption of the acylhydrazone crosslinking under high shear rates [39]. This shear-thinning behavior bestowed an injectable property on the hydrogel. It was observed that the hydrogel could be extruded through a syringe (Figure 3d, inset).

### 3.5. Thermo-Responsive and Sol–Gel Transition of Hydrogels

**Figure 4:**
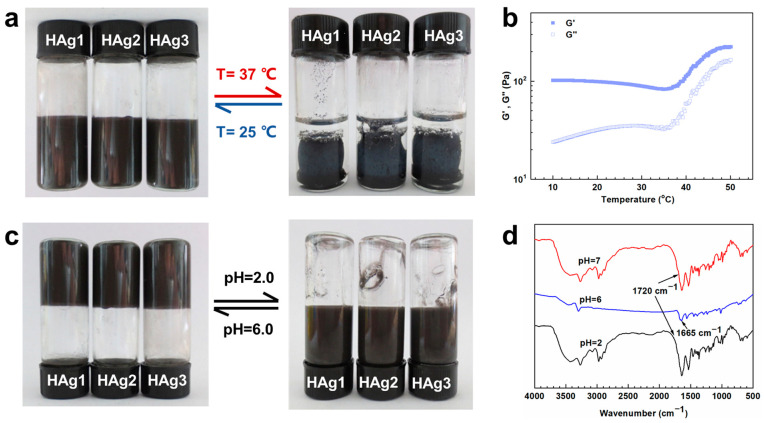
(**a**) Thermo-responsive performances of the hydrogels; (**b**) The modulus variation of HAg1 upon the increasing of temperature; (**c**) Photographs of the sol–gel transition of hydrogels; (**d**) FT-IR spectra of the mixture of the copolymer and AgNPs1 at different pH.

As the prepared copolymer exhibited a thermo-responsive property, the temperature responsiveness of its hydrogels was examined. When the hydrogels were placed at 37 °C for 5 min, it was observed that the volume of the hydrogels shrunk due to the collapsion of polymer chains [39] (Figure 4a). When the hydrogels were returned to room temperature, the hydrogels could slowly return to their original state, indicative of the thermo-responsive property of the hydrogels. Dynamic rheological measurements were performed to reveal the temperature responsiveness of the hydrogel. As depicted in Figure 4b, *G′* was invariably greater than *G″*, and the modulus increased dramatically at around 34 °C to 50 °C, suggesting that the hydrogel network was strengthened. This was mainly ascribed to the collapsion of polymer chains.

In addition, the hydrogels exhibit a reversible sol–gel transition. As shown in Figure 4c, when HCl (4 M) was added to the hydrogels to adjust the pH of the mixture to 2.0, the hydrogels transformed into free-flowing solutions. The mixture could recover hydrogels, as the pH was tuned to 6, suggesting the reversible sol–gel transition of hydrogels by altering the pH. This transition can be attributed to the network with reversible dissociation and regeneration of acylhydrazone crosslinking between hydrazides of AgNPs hybrids and the ketone groups of copolymer. As illustrated in Scheme 1, acylhydrazone was generated at a high pH, while it could dissociate at a low pH, leading to the hydrogels with a reversible sol–gel transition. FT-IR spectroscopy was used to confirm the mechanism. As shown in Figure 4d, the mixture of AgNPs1 and P_70_ did not become hydrogel at pH 7, and the absorption peak of ketone C=O appeared at 1720 cm^−1^. When the pH was adjusted to 6, the characteristic absorption peak of the ketone C=O at 1720 cm^−1^ disappeared, while the characteristic absorption peak of the acylhydrazone C=N was found at 1663 cm^−1^, indicating that acylhydrazone bond was formed. When pH was adjusted to 2, C=N at 1663 cm^−1^ disappeared, and the peak of ketone C=O was observed at 1720 cm^−1^ again, which means that the hydrazone bond dissociated.

### 3.6. Antibacterial Assay of Hydrogels

**Figure 5:**
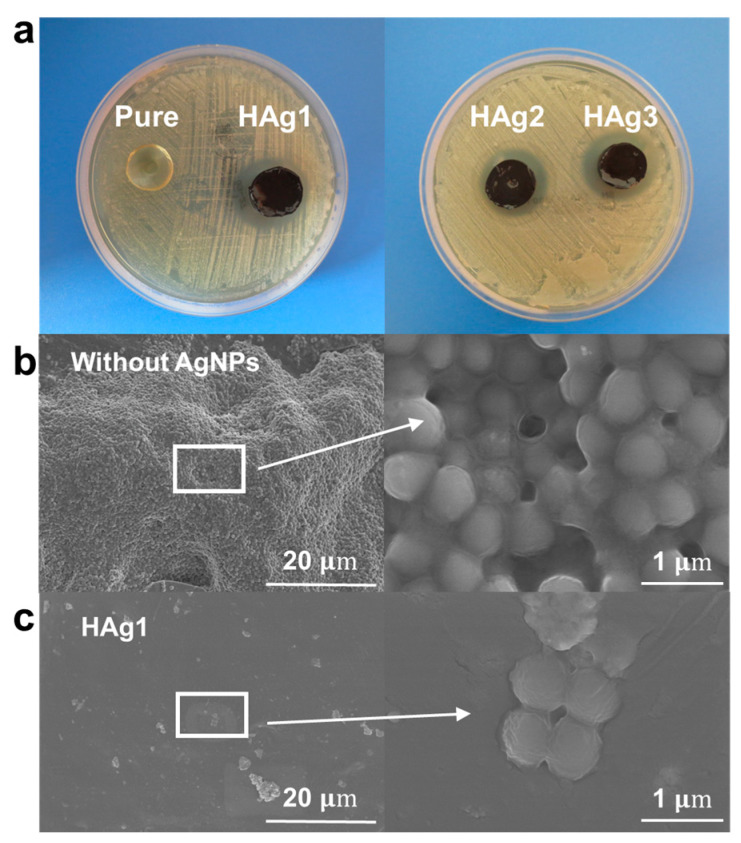
(**a**) Photographs of the inhibition zones of the hydrogels for *S. aureus*; SEM images morphology of *S. aureus* on H0 (**b**) and HAg1 (**c**).

It is well known that AgNPs have wide-spectrum antimicrobial properties [33]. Thus the antimicrobial properties of the obtained hydrogels were examined. Here, *S. aureus* was chosen as a representative bacterium, as *S. aureus* has high lethality and sturdy resistance to antibiotics and is one of the major causes of hospital-acquired infections [48]. The hydrogel (H0) of copolymer crosslinked with hexanedihydrazide rather than AgNPs hybrids served as control. As shown in Figure 5a, the inhibition zone of H0 is zero, suggesting that hydrogel without AgNPs did not have an inhibitory effect on *S. aureus*. In contrast, the inhibition zones of HAg1, HAg2 and HAg3 were 24.05 mm, 25.48 mm and 23.97 mm, respectively, which indicated that the hydrogels with AgNPs were able to kill *S. aureus* [49]. Furthermore, the morphology changes of *S. aureus* on hydrogels was observed by SEM. It can be seen that a large number of *S. aureus* grew on the H0 surface and had smooth surfaces (Figure 5b). In contrast, there were very few bacteria on the surface of the three hydrogels with AgNPs hybrids, and they show wrinkled or cracked cell surfaces (Figure 5c and Figure S10). Those results indicate that the hydrogels had good antibacterial properties. This should be attributed to Ag could be release slowly from the hydrogels and killed bacteria.

### 3.7. Conductivity and Sensing Performance of Hydrogels

**Figure 6:**
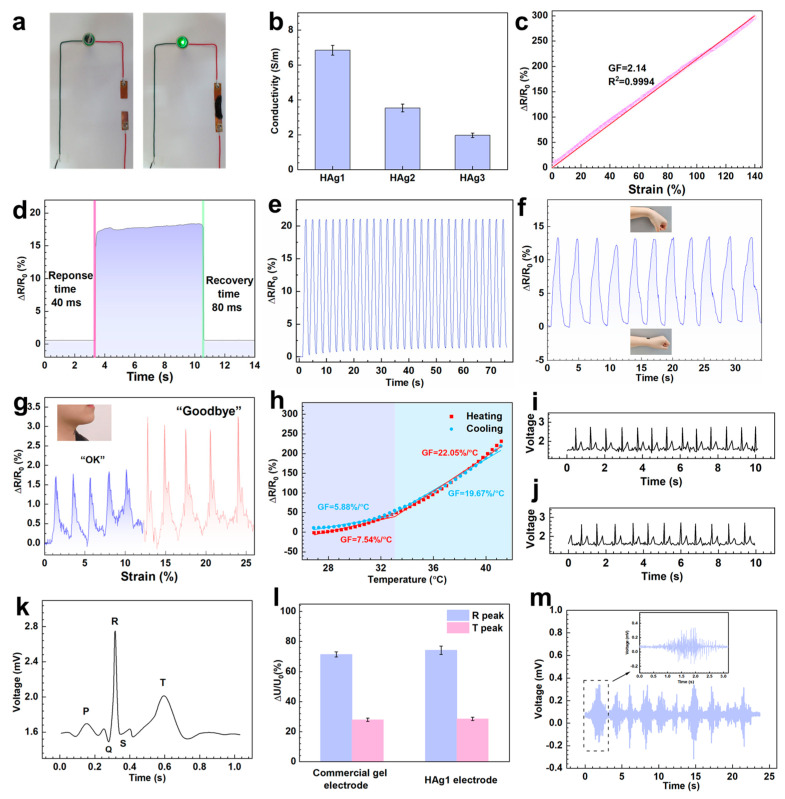
(**a**) Photographs of the series circuit with the LED connected to HAg1; (**b**) Conductivity of the hydrogels; (**c**) Relative resistance (ΔR/R_0_) of HAg1 sensor with increment of tensile strain. The red line is the fitting line; (**d**) Response time and recovery time of HAg1 sensor; (**e**) The response stability of HAg1 sensor for 30 cycles at a strain of 10%; (**f**) ΔR/R_0_ curves of the sensor with periodical bending of the wrist; (**g**) The variation of ΔR/R_0_ during speech; (**h**) ΔR/R_0_ change as a function of temperature; (**i**,**j**) Commercial Ag/AgCl gel electrodes and HAg1 electrodes for ECG signals detection; (**k**) Single ECG signal recorded by HAg1; (**l**) T/R peak ratio and baseline deviation for HAg1 electrodes and commercial Ag/AgCl gel electrodes; (**m**) EMG signals of HAg1 electrodes recorded on the extensor muscle.

In order to determine whether the prepared hydrogels with AgNPs have conductivity, a series circuit was designed. As shown in Figure 6a, when HAg1 was placed in the circuit and the LED bulb was lit, it indicated the conductivity of HAg1. The conductivity of the hydrogels was measured (Figure 6b). The conductivities of HAg1, HAg2 and HAg3 were 6.85 S/m, 3.54 S/m and 1.97 S/m, respectively. The conductivity of hydrogels should be mainly attributed to electronically conductive AgNPs, as they have high conductivity [29]. Meanwhile, the ionic conduction also existed in hydrogels due to the presence of ions such as H^⁺^ and Cl^−^ [29], which resulted from the addition of small amounts of HCl solution. Among them, HAg1 had the highest conductivity, as HAg1 contained the most AgNPs (Table S3), which is higher than that of repost hydrogels with AgNPs [33,50]. Based on its high conductivity, HAg1 was tested to use as a strain sensor. The real-time change in resistance with tensile strain was measured. As displayed in Figure 6c, the resistance rates (ΔR/R_0_) increased with the increasing of tensile strain. The curve of ΔR/R0 versus the applied strain was linearly fitted with a high-regression coefficient (R^2^). The gauge factor (GF), used to evaluate the sensitivity of the sensor [6], reached up to 2.14. Further, the hydrogel exhibited a fast response, and the response time and recovery time were 40 ms and 80 ms during the stretching and recovering processes (Figure 6d), respectively, which are less than that of human skin (~0.1 s) [51]. Apart from fast response, the sensor exhibited repeatable sensing during cyclic stretching. As shown in Figure 6e, ΔR/R_0_ changes periodically at 10% strain and remained stable after 30 cycles of the stretching–recovering process. Those results suggest that the HAg1 sensor had accurate and rapid detection of the strain signal, which may be used to monitor the human body’s movement. Thus, the sensor adhered to the volunteer’s wrist to detect its bending activities, as shown in Figure 6f. The ΔR/R_0_ values remained almost steady during the repeated bending process, indicating stable and durable sensitivity. Interestingly, the strain sensor could detect the characteristic signals of speech when it was attached to the throat, When the volunteer said “OK” and “goodbye” repeatedly, the distinguishable and reproducible signals were recorded (Figure 6g), indicating that the HAg1 sensor can be potentially utilized for speech recognition.

Apart from strain sensing, the hydrogel exhibited a temperature detection capability due to the temperature responsiveness of the hydrogel. Figure 6h shows the temperature/resistance relation of HAg1 between 27 °C and 41 °C. The ΔR/R_0_ values had a positive correlation with the temperature, i.e., the resistance increased with the increment of temperature. This should result from AgNPs being gradually wrapped by the collapsed polymeric chains after temperature responsiveness, which impeded electron transport and led to the increasing of resistance. Based on the ΔR/R_0_ versus temperature, there are two temperature GFs, i.e., the GF values are 7.54%/°C and 22.05%/°C during the temperature ranges of 27~33 °C and 33~41 °C, respectively, which is higher than that of the previously reported temperature-sensing materials [52,53]. Moreover, the ΔR/R_0_ values at the cooling process mostly overlapped with that of heating, and the temperature GFs during cooling were also close to that of heating, suggesting that temperature sensing was reversible. In comparison with the previously reported hydrogels crosslinked with dynamic covalent bonds or containing AgNPs [54,55,56,57] (Table S4), HAg1 simultaneously exhibited antimicrobial activity, high conductivity and thermomechanical sensing.

Furthermore, the hydrogel was used as the electrode for ECG signal detection. Here, a commercial Ag/AgCl gel electrode was used as control. HAg1 electrode pairs were attached to the human skin, and ECG signals were recorded continuously (Figure 6j), which was similar to that of a commercial electrode (Figure 6i). Compared with that of a commercial electrode (Figure S11), the P wave, QRS complex and T wave of the ECG signal were more clearly detected by HAg1 hydrogel (Figure 6k). Moreover, the ratio of T and R peak values (T/R ratio) was closer to 1/3 (Figure 6l), indicating good quality for sensing [26]. The results showed that the HAg1 hydrogel electrodes were better able to detect the ECG signals in comparison with commercial gel electrodes. Furthermore, HAg1 could also serve as electrodes to record EMG signals when the volunteer gripped an object (5 kg) (Figure 6m).

## 4. Conclusions

We prepared well-defined thermo-responsive copolymer P(NIPAM-co-DAAM-co-AM) by RAFT polymerization and synthesized AgNPs with hydrazide groups by surface functionalization and hydrazinolysis. The hybrid hydrogels were generated by mixing the copolymer and AgNPs via the formation of acylhydrazone bonds between ketone groups and hydrazides. Based on dynamical acylhydrazone crosslinking, the obtained hydrogels exhibited sol–gel transition, self-healable and injectable properties. And the hydrogels displayed thermo-responsive behavior. Moreover, the hydrogels had antimicrobial properties and high conductivity (6.85 S/m). The fabricated hydrogel-based sensors exhibited strain and temperature sensing, and the sensors would detect human motion and speech. They also could be used as electrodes for ECG and EMG signals detection. With these attractive characteristics, the dynamic hydrogels may find potential applications in antibacterial wearable electronics, health monitoring and speech recognition.

## Data Availability

The original contributions presented in this study are included in the article/Supplementary Material. Further inquiries can be directed to the corresponding authors.

## Supplementary Materials

The following supporting information can be downloaded at: https://www.mdpi.com/article/10.3390/polym17010054/s1, Table S1: Feeding ratio of AgNPs with hydrazide bonds. Table S2: Molecular weight, polydispersity of the P70 measured by GPC. Table S3: Components of hydrogels; Table S4: Performances and functions of our hydrogels with AgNPs compared with the previously reported hydrogels with dynamic covalent bonds or containing AgNPs. Figure S1: (a) 1H NMR spectrum of P70 in D2O; (b) GPC traces of P70. Figure S2: Temperature dependence of optical transmittance for aqueous solution of P70. The insets images are P70 at 30 °C and 40 °C, respectively. Figure S3: SEM images of HAg2 (a) and HAg3 (b). Figure S4: EDS diagrams of silver nanoparticle hydrogels: (a) HAg1, (b) HAg2, (c) HAg3. Figure S5: FT-IR spectra of the polymer, AgNPs1 and HAg1. Figure S6: XRD patterns of the hydrogels. Figure S7: XRD patterns of the hydrogels after storing at room temperature for 1 month. The hydrogels were kept at room temperature under seal (wrapped with plastic wrap). Figure S8: Stress–strain curves of the original and the self-healed hydrogels. Figure S9: (a) Stress–strain curves of the hydrogels and the self-healed hydrogels after storing at room temperature for two weeks, and (b) the corresponding self-healing efficiencies. The hydrogels were kept at room temperature under seal (wrapped with plastic wrap). Figure S10: SEM images of the S. aureus morphology on hydrogels for 6 h: (a) HAg2, (b) HAg3. Figure S11: Single ECG signal recorded by commercial Ag/AgCl gel electrodes.
