# Supplementary material for: Self-Healable, Antimicrobial and Conductive Hydrogels Based on Dynamic Covalent Bonding with Silver Nanoparticles for Flexible Sensor
Source: Polymers (Basel). 2024 Dec 29;17(1):54. doi: 10.3390/polym17010054 (PMC11723201; doi:10.3390/polym17010054)
Supplement: Supplementary file 1 [file polymers-17-00054-s001.zip › polymers-3383931-supplementary.pdf]

Supplementary information

Self-healable, Antimicrobial and Conductive Hydrogels based on Dynamic Covalent Bonding with Silver Nanoparticles for Flexible Sensor

Te Qi[1], Xuefeng Liu[1], Nan Zheng[1], Jie Huang[1], Wenlong Xiang[1], Yujin Nie[1], Zanru Guo*[1] and Baixue Cai*[2]

[1]College of Chemistry, Chemical Engineering and Environmental Science, Minnan Normal University, Zhangzhou 363000, P. R. China.

[2]Chongqing Academy of Metrology and Quality Inspection, Chongqing 401120, PR China.

Table S1. Feeding ratio of silver nanoparticles with hydrazide bonds

Sample	Mass (g)	Mass (g)	Mass (g)
	$AgNO_3$	Methyl 3-mercaptopropionate	Hydrazine hydrate
AgNPs1	0.8500	0.1200	0.2300
AgNPs2	0.8500	0.2400	0.4610
AgNPs3	0.8500	0.3600	0.6920

Table S2. Molecular weight, polydispersity of the P_{70} measured by GPC

Polymers	DP_n[a]	$M_{n,theo}$[b]	$M_{w,GPC}$[c]	$M_{n,GPC}$[c]	PDI[c]
P_{70}	800	88160	96326	64967	1.483

[a] DP_n refer to the mole ratio of monomer/initiator, [b] Expected number-average molecular weight from polymerization stoichiometry, [c] Determined by CPC in DMF

Table S3. Components of hydrogels

Sample	AgNPs (g)	P_{70} (g)	Water (g)
HAg1	1.66 (AgNPs1)	2.0	32.94
HAg1	1.11 (AgNPs2)	2.0	27.99
HAg1	0.85 (AgNPs3)	2.0	25.65

Table S4. Performances and functions of our hydrogels with AgNPs compared with the previously reported hydrogels with dynamic covalent bonds or containing AgNPs (Yes stands for having the feature; No stands for not having the feature).

Refs.	Mechanical Properties (Fracture strength)	Antimicrobial ability	Conductivity (S/m)	Strain sensing (GF)	temperature-sensing
**This work**	**78.2 Pa**	**Yes**	**6.85**	**2.14**	**Yes**
[1]	0.186 MPa	Yes	—	—	No
[2]	41.0 kPa	No	—	5.9	No
[3]	0.91 MPa	No	1.31	2.06-11.34	No
[4]	6.0 MPa	No	0.31	6.6	Yes
[5]	0.12 MPa	Yes	0.31	2.01-4.73	No
[6]	0.69 MPa	No	0.11	2.49-8.65	No

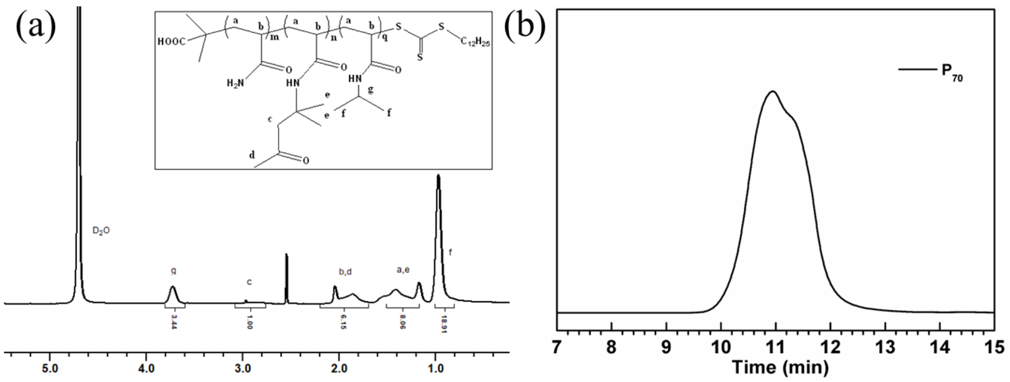

Figure S1. (a) ^{1}H NMR spectrum of P_{70} in D_2O; **(b)** GPC traces of P_{70}

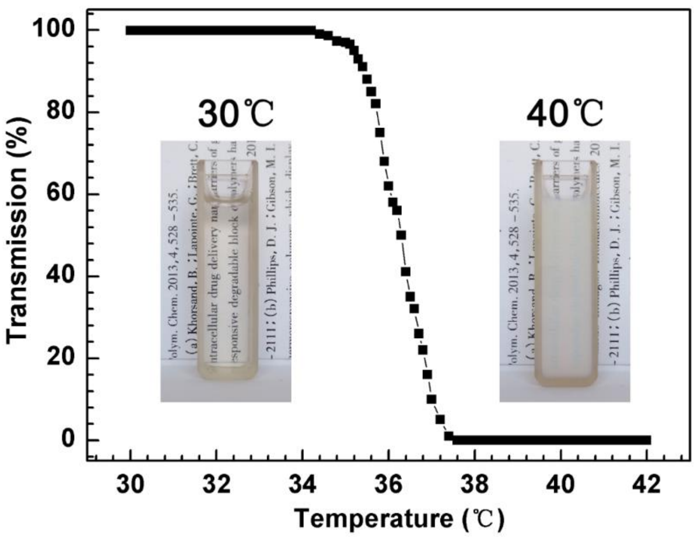

Figure S2. Temperature dependence of optical transmittance for aqueous solution of P_{70}. The insets images are P_{70} at 30 °C and 40 °C, respectively.

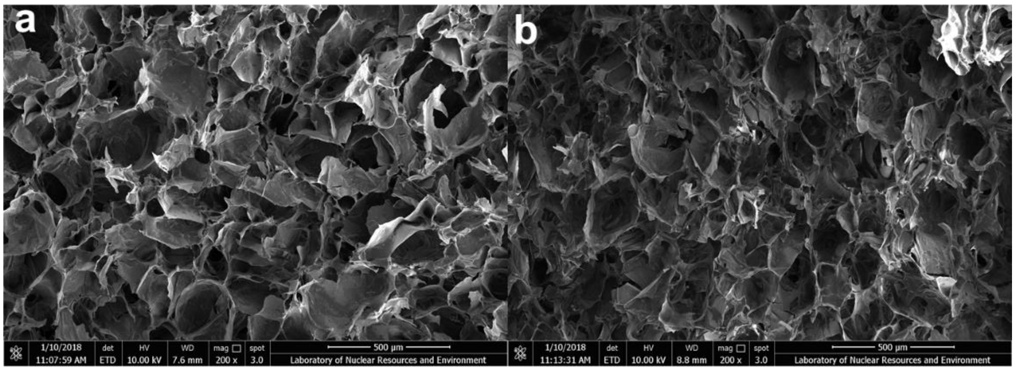

Figure S3. SEM images of HAg2 (a) and HAg3 (b).

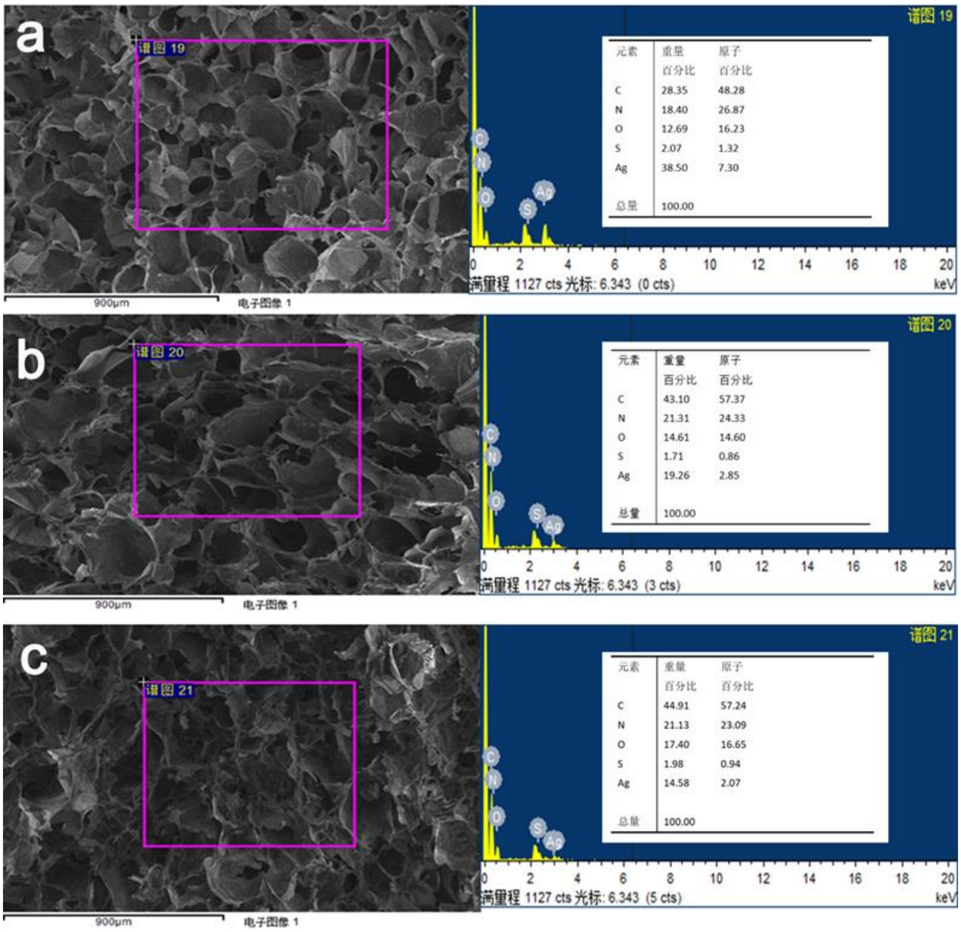

Figure S4. EDS diagrams of silver nanoparticle hydrogels: (a) HAg1, (b) HAg2, (c) HAg3.

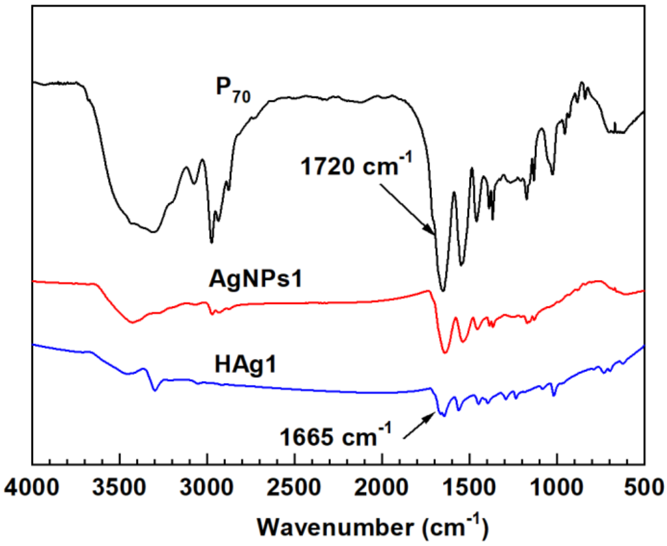

Figure S5. FT-IR spectra of the polymer, AgNPs1 and HAg1.

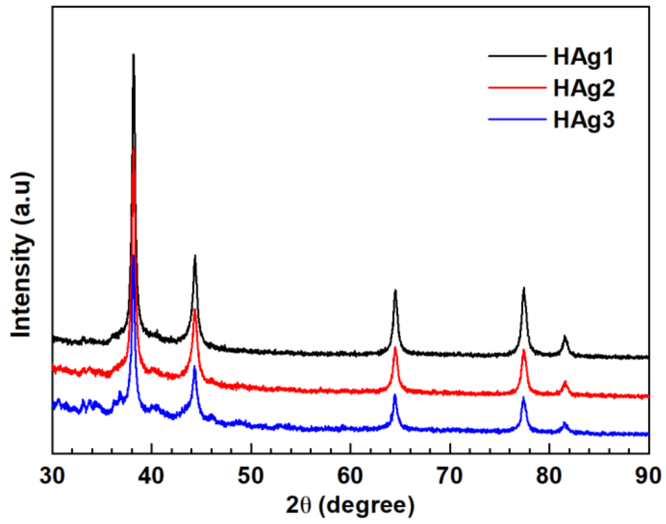

Figure S6. XRD patterns of the hydrogels.

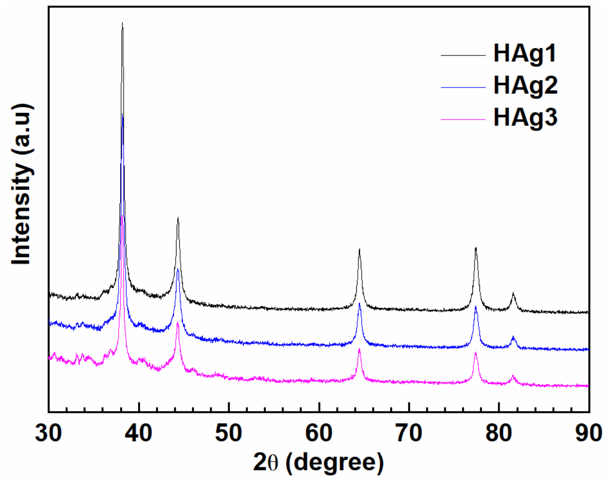

Figure S7. XRD patterns of the hydrogels after storing at room temperature for 1 month. The hydrogels were kept at room temperature under seal (wrapped with plastic wrap).

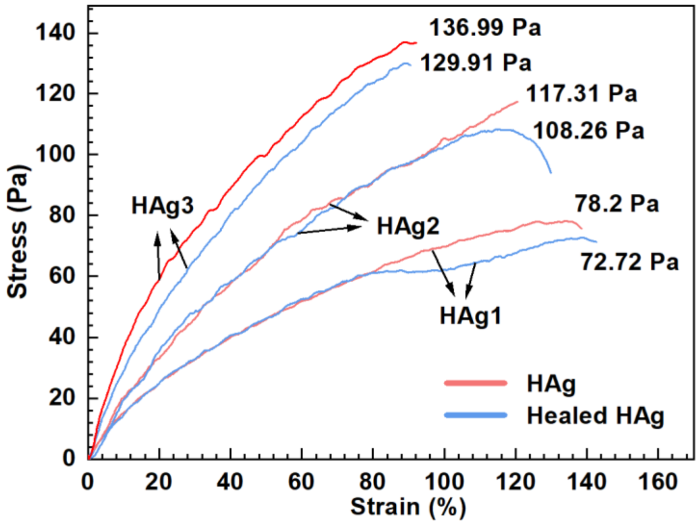

Figure S8. Stress-strain curves of the original and the self-healed hydrogels.

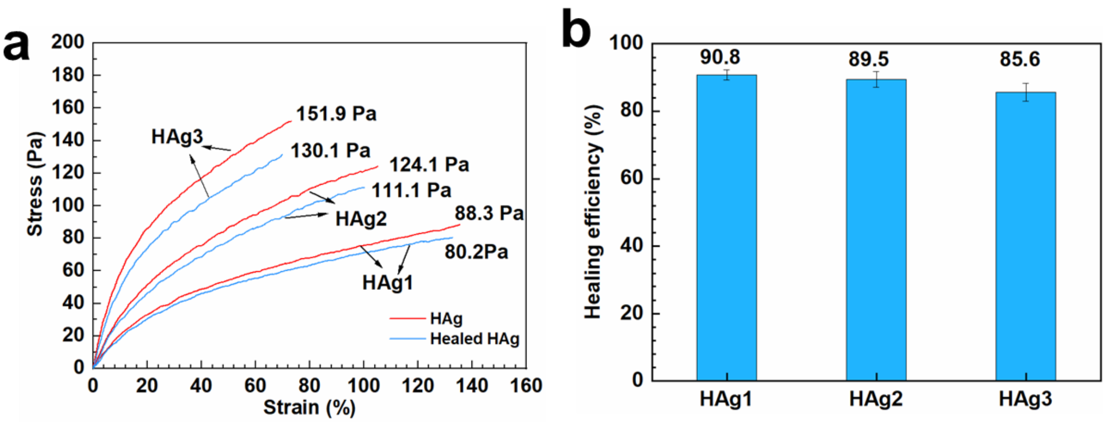

Figure S9. (a) Stress-strain curves of the hydrogels and the self-healed hydrogels after storing at room temperature for two weeks, and (b) the corresponding self-healing efficiencies. The hydrogels were kept at room temperature under seal (wrapped with plastic wrap).

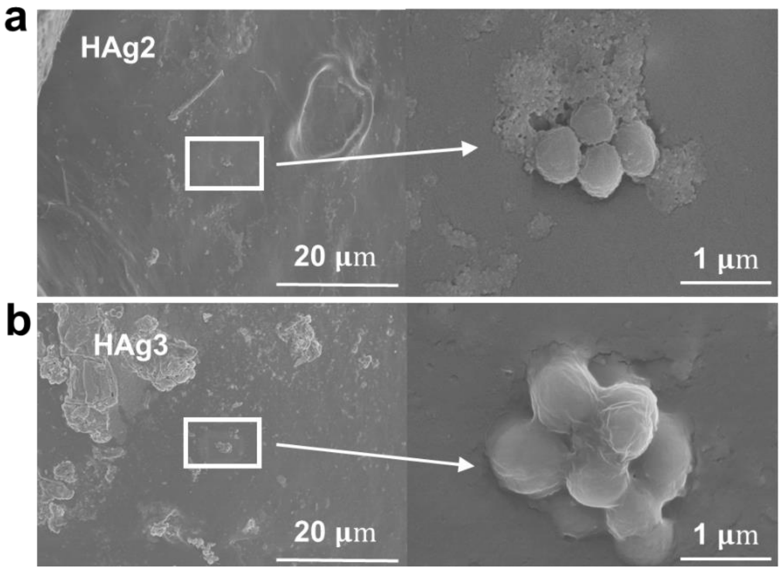

Figure S10. SEM images of the *S. aureus* morphology on hydrogels for 6 h: (a) HAg2, (b) HAg3.

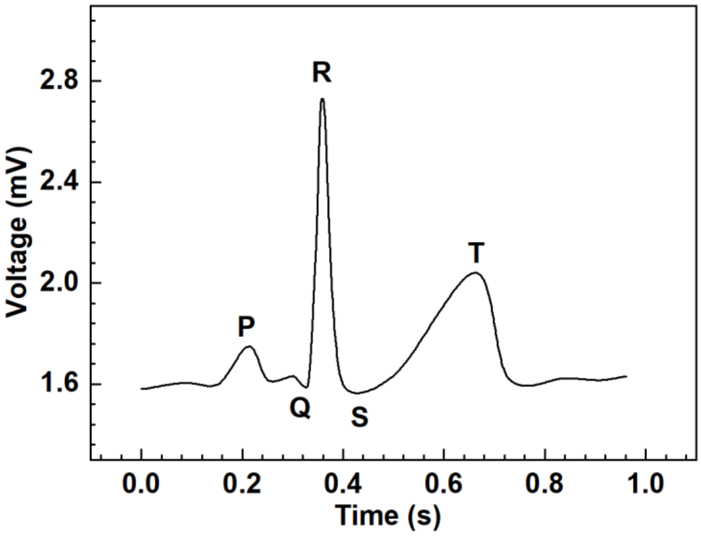

Figure S11. Single ECG signal recorded by commercial Ag/AgCl gel electrodes.

References

[1] Ren, Z.; Ke, T.; Ling, Q.; Zhao, L.; Gu, H. Rapid self-healing and self-adhesive chitosan-based hydrogels by host-guest interaction and dynamic covalent bond as flexible sensor. *Carbohydr. Polym.* **2021**, 273, 118533.

[2] Liu, X.; Ren, Z.; Liu, F.; Zhao, L.; Ling, Q.; Gu, H. Multifunctional self-healing dual network hydrogels constructed via host–guest interaction and dynamic covalent bond as wearable strain sensors for monitoring human and organ motions. *ACS Appl. Mater. Interfaces* **2021**, 13, 14612-14622.

[3] Shekh, M. I.; Zhu, G.; Xiong, W.; Wu, W.; Stadler, F. J.; Patel, D.; Zhu, C. Dynamically bonded, tough, and conductive MXene@oxidized sodium alginate: Chitosan based multi-networked elastomeric hydrogels for physical motion detection. *Int. J. Biol. Macromol*. **2023**, 224, 604-620.

[4] Liu, H.; Wang, X.; Cao, Y.; Yang, Y.; Yang, Y.; Gao, Y.; Ma, Z.; Wang, J.; Wang, W.; Wu, D. Freezing-tolerant, highly sensitive strain and pressure sensors assembled from ionic conductive hydrogels with dynamic cross-links. *ACS Appl. Mater. Interface*. **2020**, 12, 25334-25344.

[5] Wang, X.; Wang, Z.; Wang, X.; Shi, L.; Ran, R. Preparation of silver nanoparticles by solid-state redox route from hydroxyethyl cellulose for antibacterial strain sensor hydrogel. *Carbohydr. Polym.* **2021**, 257, 117665.

[6] Fan, Q.; Nie, Y.; Sun, Q.; Wang, W.; Bai, L.; Chen, H.; Yang, L.; Yang, H.; Wei, D. Nanocomposite hybrid biomass hydrogels as flexible strain sensors with self-healing ability in harsh environments. *ACS Appl. Polym. Mater*. **2022**, 4, 1626-1635.
